# Synovial phenotypes in rheumatoid arthritis correlate with response to biologic therapeutics

**DOI:** 10.1186/ar4555

**Published:** 2014-04-30

**Authors:** Glynn Dennis, Cécile TJ Holweg, Sarah K Kummerfeld, David F Choy, A Francesca Setiadi, Jason A Hackney, Peter M Haverty, Houston Gilbert, Wei Yu Lin, Lauri Diehl, S Fischer, An Song, David Musselman, Micki Klearman, Cem Gabay, Arthur Kavanaugh, Judith Endres, David A Fox, Flavius Martin, Michael J Townsend

**Affiliations:** 1 Departments of Immunology Discovery, Genentech, South San Francisco, California, USA; 2 ITGR Diagnostics Discovery, Genentech, South San Francisco, California, USA; 3 Bioinformatics and Computational Biology, Genentech, South San Francisco, California, USA; 4 Non-clinical Biostatistics, Genentech, South San Francisco, California, USA; 5 Pathology, Genentech, South San Francisco, California, USA; 6 Bioanalytical Sciences, Genentech, South San Francisco, California, USA; 7 Product Development, Genentech, South San Francisco, California, USA; 8 University Hospital of Geneva, Geneva, Switzerland; 9 University of California San Diego, San Diego, California, USA; 10 Rheumatic Disease Core Center and Division of Rheumatology, Department of Internal Medicine, University of Michigan Medical School, Ann Arbor, Michigan, USA; 11 Current address: Inflammation Therapeutic Area, Amgen, 1201 Amgen Court West, Seattle, Washington, USA

## Abstract

**Introduction:**

Rheumatoid arthritis (RA) is a complex and clinically heterogeneous autoimmune disease. Currently, the relationship between pathogenic molecular drivers of disease in RA and therapeutic response is poorly understood.

**Methods:**

We analyzed synovial tissue samples from two RA cohorts of 49 and 20 patients using a combination of global gene expression, histologic and cellular analyses, and analysis of gene expression data from two further publicly available RA cohorts. To identify candidate serum biomarkers that correspond to differential synovial biology and clinical response to targeted therapies, we performed pre-treatment biomarker analysis compared with therapeutic outcome at week 24 in serum samples from 198 patients from the ADACTA (ADalimumab ACTemrA) phase 4 trial of tocilizumab (anti-IL-6R) monotherapy versus adalimumab (anti-TNFα) monotherapy.

**Results:**

We documented evidence for four major phenotypes of RA synovium – lymphoid, myeloid, low inflammatory, and fibroid - each with distinct underlying gene expression signatures. We observed that baseline synovial myeloid, but not lymphoid, gene signature expression was higher in patients with good compared with poor European league against rheumatism (EULAR) clinical response to anti-TNFα therapy at week 16 (*P* =0.011). We observed that high baseline serum soluble intercellular adhesion molecule 1 (sICAM1), associated with the myeloid phenotype, and high serum C-X-C motif chemokine 13 (CXCL13), associated with the lymphoid phenotype, had differential relationships with clinical response to anti-TNFα compared with anti-IL6R treatment. sICAM1-high/CXCL13-low patients showed the highest week 24 American College of Rheumatology (ACR) 50 response rate to anti-TNFα treatment as compared with sICAM1-low/CXCL13-high patients (42% versus 13%, respectively, *P* =0.05) while anti-IL-6R patients showed the opposite relationship with these biomarker subgroups (ACR50 20% versus 69%, *P* =0.004).

**Conclusions:**

These data demonstrate that underlying molecular and cellular heterogeneity in RA impacts clinical outcome to therapies targeting different biological pathways, with patients with the myeloid phenotype exhibiting the most robust response to anti-TNFα. These data suggest a path to identify and validate serum biomarkers that predict response to targeted therapies in rheumatoid arthritis and possibly other autoimmune diseases.

**Trial registration:**

ClinicalTrials.gov NCT01119859

## Introduction

Rheumatoid arthritis (RA) is an autoimmune disease characterized by symmetrical joint involvement, inflammation, synovial lining hyperplasia, and formation of invasive granulation tissue or pannus. Progression of RA pathogenesis is associated with impaired joint function resulting from immune-mediated destruction of bone and cartilage [1-3]. Considerable patient-to-patient variation exists in the number of affected joints, the levels of autoantibody titers and serum cytokines, and the rate of joint destruction [4,5]. Disease heterogeneity is further evident upon histological examination of synovial tissues, where a spectrum of cellular compositions are found, ranging from diffuse leukocytic infiltration to well-organized, lymphocyte-containing follicle-like structures [6].

Not surprisingly, RA is also heterogeneous in response to treatment. Although the development of targeted therapeutic strategies blocking TNF α, IL-6 receptor, T-cell co-stimulation blockade and B-cell depletion have provided meaningful clinical benefit to patients, a key unmet need in the management of RA is the prospective identification of patients who are likely to benefit from specific therapies. We hypothesized that a deeper understanding of the molecular basis of disease heterogeneity will lead to the discovery of predictive biomarkers able to identify individual patients who will benefit from a particular therapeutic strategy [7].

Insight into pathogenic molecular pathways of RA has emerged in recent years from genome-wide analysis of synovial tissue gene expression. Multiple studies have assessed molecular heterogeneity in RA tissue, but few findings have been validated with subsequent cohorts. Early studies [8,9] revealed considerable molecular heterogeneity and proposed RA patient subgroups exhibiting gene expression patterns consistent with ongoing inflammation and adaptive immunity or, alternatively, little immune infiltrate and instead expressing sets of genes involved in extracellular matrix remodeling [10]. Further, it has been observed that lymphoid follicle-containing synovial samples have increased expression of sets of genes involved in Janus kinase (JAK)/signal transducer and activator of transcription (STAT) signaling, and IL-7 signal transduction [11], suggesting that differences in gene expression patterns reflect differences in relative cellular composition of the RA joint.

Gene and protein expression studies of synovial tissue at baseline prior to initiating TNFα blockade have also generated different hypotheses to account for the differences between good and poor responders. In two studies, patients who responded to anti-TNFα treatment had transcription profiles enriched for inflammatory processes and TNFα protein expression [12,13], whereas another report concluded that good responders actually had lower inflammatory processes and cell-surface markers such as the IL-7 receptor alpha chain [14]. A large gene expression study of synovial tissues from 62 patients obtained prior to initiating anti-TNFα therapy identified very few transcripts that were different between good and poor responders [15]. In the current study, we build on these observations by characterizing different molecular phenotypes of RA synovium - lymphoid, myeloid and fibroid - and used these to identify soluble biomarkers that predict differential treatment effects in RA patients.

## Methods

### Patients and synovial tissues

Synovial tissues were obtained from RA subjects undergoing arthroplasty and/or synovectomy of affected joints (University of Michigan, two sequential cohorts, n = 49 and n = 20). Written consent was obtained from patients, and the University of Michigan Institutional Review Board approved the study protocol. RA was diagnosed based upon the 1987 College of Rheumatology (ACR) criteria [16]. Patients were treated using the standard of care for RA (non-steroidal anti-inflammatory drugs (NSAIDs) and disease-modifying anti-rheumatic drugs (DMARDs)) and some patients were also treated with biologics (adalimumab, etanercept, infliximab, anakinra and rituximab). Patients were diagnosed with RA at least three years before surgery and 70% of patients for whom data were available were rheumatoid factor (RF)-positive. Excised tissues were immediately snap-frozen in liquid nitrogen and stored at -80°C. Each tissue was used for both histology and RNA extraction. For cryo-sectioning, samples were brought briefly to -20°C, sectioned and immediately returned to -80°C to maintain RNA integrity. All tissues used for downstream studies were prospectively randomized during processing and sectioning, prior to expression analysis, to minimize technical batch effects in the data.

### RNA isolation

Frozen samples were weighed and homogenized in RLT buffer (Qiagen, Valencia, California, USA) + β-mercaptoethanol (10 μl/ml) at a concentration of 100 mg/ml. Prior to isolating RNA using an RNeasy minikit (Qiagen) with on-column DNase digestion, samples were digested with Proteinase K (Qiagen) for 10 minutes at 55°C.

### Histopathology and immunohistochemistry

Stains were performed on 5-μm-thick frozen sections of human synovial tissue fixed in acetone. Some sections were stained with hematoxylin and eosin for histologic evaluation. Other sections were blocked in 10% serum for 30 minutes and stained for the detection of cells expressing the following lineage markers (CD20 - mouse anti-human clone L26, 5 μg/ml, Dako (Carpinteria, California, USA); CD3 - rabbit anti-human antibody SP7, 1:200 dilution, NeoMarkers (Fremont, California, USA) and CD68 - mouse anti-human clone KP-1, 2.5 μg/ml, Dako). All immunohistochemical stains were detected with species specific, biotinylated secondary antibodies and 3,3′-diaminobenzidine (DAB).

### Microarray hybridization

The protocols for preparation of cRNA and for array hybridization were followed as recommended by Affymetrix, Inc. (Santa Clara, CA, USA). Samples were hybridized to GeneChip® Human Genome U133 Plus 2.0 Arrays (Affymetrix, Inc.). Arrays were washed and stained in the Affymetrix Fluidics station and scanned on a GeneChip® scanner 3000. Expression signals were obtained using the Affymetrix GeneChip® operating system and analysis software.

### Microarray data analyses

Microarray data for all samples are freely available for download [GEO:GSE48780] [17,18]. Statistical analysis of microarray data was performed with the open-source tools available in the statistical programming environment R [19] and the Bioconductor project [20]. Microarray data was normalized using the robust multichip average method (RMA) [21,22]. This approach included three steps: background correction, quantile normalization and summarization. Following RMA processing, probe sets were filtered to exclude those that are believed to cross-hybridize or show other deficiencies according to the Affymetrix quality assessment classification (only *A-class* probes were included). In addition, probe sets without an Entrez ID-mapping were excluded. Microarray data were further filtered to a single probe set per gene. For genes with multiple probesets, only the probe set with the largest variance was used [23].

For the primary analysis of the University of Michigan samples, probe sets were further filtered retaining the top 40% most variable genes based on their SD across all samples [24]. Probe sets were then centered and scaled. In order to identify groups of samples that showed similar expression profiles, we used agglomerative hierarchical clustering (Ward’s method, Euclidean distance on scaled and centered data). We divided the samples into groups based on the resulting clustering. The optimal number of groups was selected via two common metrics that quantify the tightness of clustering by considering the distance between samples within a group and the inter-group distance: mean silhouette width and k-nearest neighbor distances. We calculated these metrics for between three and eight groups, and both metrics indicated that separating the samples into five groups minimized the within-group sample distance and maximized the between-group distance. For testing cluster robustness, we used a re-sampling approach in which we randomly excluded five samples from the dataset, then selected the top 40% highest variance genes and performed clustering using the partitioning around medoids (PAM) algorithm with *k* = 5*.* The frequency with which a pair of samples was found in the same cluster in a given re-sampling was calculated for all pairs. Significantly over-represented pathways between the phenotypes were identified using the Database for Annotation, Visualization and Integrated Discovery (DAVID) tool [25]. For each phenotype a set up-regulated and a separate set of downregulated genes was identified by comparing samples from that phenotype with all other samples and selecting genes that were differentially expressed at a false discovery rate (FDR) cutoff of 0.01. These differentially expressed sets were used as input to the DAVID tool using the default parameters recommended by the developers. Outputs from the DAVID analysis, including levels of genes from each process within the four synovial groups as defined by their *t*-statistic values and *P*-values, are available in the Additional files 1 and 2. The external dataset GSE21537 was downloaded from the GEO database, and was normalized and background-corrected using the variance stabilization and normalization (VSN) for microarray.

### Gene set analysis

Pathway level analysis was carried out using gene set enrichment analysis (GSEA), using the Bioconductor GSEAlm package [26]. Gene sets used in the analysis comprised the Molecular Signatures DataBase (MSigDB) from the Broad Institute [27], purified immune-cell type-specific gene expression [28], and a manually curated list of genes associated with angiogenesis processes. In addition, gene sets were defined based upon gene expression from microarray analysis of *in vitro* stimulated sorted blood monocytes (CD14^+^) that underwent classical activation (M1) with lipopolysaccharide (LPS) and IFNγ versus alternative activation (M2) with IL-4 and IL-13 for 24 hours, as well as *in vitro* stimulation of primary synovial fibroblasts from RA patients with TNFα or media-only control for 6 hours. All genes in each of the gene sets are listed in Additional file 3: Table S1. Summary gene-set scores were calculated using a quartile trimmed mean of the normalized probe-set values present in the gene set. Statistical significance of gene-set scores between the different synovial phenotypes was calculated using the *t*-test followed by Benjamini-Hochberg correction of *P*-values [29].

Group-specific genes for the myeloid, lymphoid and fibroid phenotypes were defined by identification of genes that were differentially expressed between each pair of groups using a moderated t-statistic (FDR <0.01), and then a list of genes was assembled for each group of the genes that were upregulated between that group and one or more others. Any gene that was differentially expressed between more than one pair of groups was discarded and the top 100 upregulated genes for each group were selected based on *P*-value ranking. Genes are listed in Additional file 3: Table S1. To assess relationships between the group-specific gene sets and response to anti-TNFα treatment, each group-specific gene set was mapped to the microarray expression dataset generated by [15] utilizing all available matching genes. Receiver-operating characteristic (ROC) analysis was performed using continuous gene-set scores compared against the European League Against Rheumatism (EULAR) good-versus-poor response criteria to anti-TNFα treatment, and area under the ROC curve (AUC) was determined for each gene set.

### Serum biomarker assessments in the ADalimumab ACTemrA (ADACTA) clinical trial

Serum samples from 198 of the 326 patients in the ADACTA trial (ClinicalTrials.gov Identifier: NCT01119859) [30], where written consent had been given for exploratory biomarker analysis, were assessed for baseline pre-treatment levels of soluble intercellular adhesion molecule 1 (sICAM1) and C-X-C motif chemokine 13 (CXCL13) using customized electrochemiluminescence assays incorporating sample diluent blocking reagents to minimize interference from heterophilic antibodies. Biomarker subgroups were defined as low (below pretreatment median) or high (equal to or greater than pretreatment median) for each of the two markers. Relative treatment effectiveness (week-24 ACR50 criteria) of adalimumab compared with tocilizumab was assessed by logistic regression for each biomarker-defined subgroup. An odds ratio >1.0 and <1.0 than one correspond to favorable outcomes for adalimumab or tocilizumab respectively. Subpopulation treatment effect pattern plot (STEPP) analysis [31] was also performed on relative treatment effectiveness (week-24 ACR50 response) of adalimumab compared with tocilizumab for these two biomarkers. Assessment of statistical significance between subgroups was assessed using the Fisher exact test. ROC analysis was performed using continuous serum biomarker values compared against achievement of ACR50 response at 24 weeks for adalimumab or tocilizumab, and the AUC was determined.

## Results

### Molecular phenotypes in RA synovium

**Figure 1:**
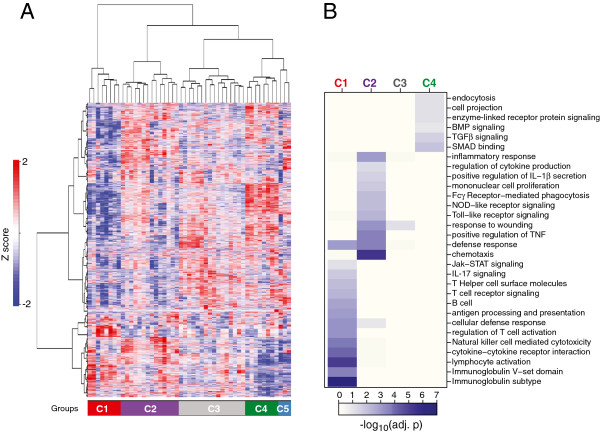
**Stratification of rheumatoid arthritis (RA) transcriptional heterogeneity into homogeneous molecular phenotypes.(A)** Two-dimensional hierarchical clustering of approximately 7,000 probes (rows), representing quantile-normalized and scaled expression values of the top 40% most variable probe sets (variability assessed using SD), in 49 RA patients (columns) inferring five molecular subgroups of synovial tissues. Patient-sample ordering and dendrogram based on agglomerative hierarchical clustering (Ward method): resulting tree used to select patient subgroups; number of patient subgroups selected to maximize mean silhouette width and *k*-nearest neighbor distances (*k* = 5 considered optimal). *z*-score-based color intensity scale for each probe in each sample is shown. Patient samples clustering into five main branches are color-coded left to right (bottom of the heatmap): C1 = red (n = 8), C2 = purple (n = 14), C3 = gray (n = 16), C4 = green (n = 8), C5 = light blue (n = 3). **(B)** Heatmap depicting over-represented Database for Annotation, Visualization and Integrated Discovery biological process categories for genes upregulated in the four largest synovial clusters. Each column represents one cluster (C1 to C4), color-coordinated as in panel **A**. Each row corresponds to a biological process category. Heatmap colors reflect log10 (adjusted *P*-value) from modified Fisher exact test for categorical over-representation. Annotation for each cluster based on the key biological processes is indicated. BMP, bone morphogenetic protein; TGF, transforming growth factor; SMAD, Sma Mothers Against Decapentaplegic; NOD, nucleotide-binding oligomerization domain; JAK-STAT, Janus kinase-signal transducer and activator of transcription.

Gene expression profiles of synovial tissues from 49 subjects with clinically diagnosed RA were subjected to unsupervised hierarchical clustering (HCL) in order to assess transcriptional heterogeneity and identify putative phenotypes of RA. We identified five main clusters of patient samples (C1 to C5) (Figure 1A)*.* These clusters were visualized using principal components analysis of the scaled and centered data (Additional file 4: Figure S1A) and samples from clusters C1 to C4 showed differences along principal components 1 and 2, whereas samples from C5 were not well-separated in these two projections. We further assessed cluster robustness using several additional statistical methods (discussed in Additional file 4: Figure S1B and C) that further confirmed C5 was not well-separated and distinct from C4. We therefore conducted all further analyses on clusters C1 to C4.

To characterize putative phenotypes of RA according to their pathway composition we first identified subsets of genes that were specifically upregulated within each of the four clusters using a one-versus-all approach (see Methods). Each of the cluster-specific gene lists was then subjected to keyword over-representation analysis using DAVID. Immune response genes were abundant in both C1 (now termed the lymphoid phenotype) and C2 (myeloid phenotype), with the C1 lymphoid gene sets highly restricted to B and/or T lymphocyte activation and differentiation, immunoglobulin production, and antigen presentation together with enrichment of cytokine signaling including the Jak/STAT pathway and IL-17 signaling (Figure 1B). In contrast, the gene sets upregulated in the C2 myeloid group were also enriched for immune function, but were characterized by processes associated with chemotaxis, TNFα and IL-1β production, Toll-like receptor and nucleotide-binding oligomerization domain (NOD)-like receptor signaling, Fcγ-receptor-meditated phagocytosis, and proliferation of mononuclear cells. Cluster 3 (designated a low inflammatory phenotype) showed only enrichment for inflammatory response and wound response processes. The remaining C4 cluster, designated the fibroid phenotype, was enriched for genes associated with transforming growth factor (TGF) β signaling, bone morphogenetic protein (BMP) signaling together with associated Sma Mothers Against Decapentaplegic (SMAD) binding, as well as endocytosis and cell projection processes (Figure 1B) but lacked enrichment of any immune system processes. We further confirmed that the identified processes of interest were not solely driven by a small set of recurring genes, by directly comparing each gene set identified by the DAVID analysis with each other and observing that their overlap was generally low (Additional file 5: Figure S2). However, these analyses also suggested certain biological processes might reflect similar gene expression profiles occurring together in the same patients, for example, Toll-like receptor signaling, NOD-like receptor signaling, and Fc-γR-mediated phagocytosis occurred together primarily in the myeloid group, whereas processes such as antigen processing and presentation overlapped with both lymphoid group processes such as B and T cell activation and myeloid group processes such as FcγR-mediated phagocytosis and mononuclear cell proliferation, as might be expected based upon their connected immunological roles. Further, examination of genes that were specifically downregulated within each of the four clusters indicated the C4 fibroid cluster had significant downregulation of multiple immune-system processes associated with B cells, immunoglobulins, myeloid cells, innate immune response, including NOD-like receptor signaling, and chemotactic processes (Additional file 6: Figure S3A). In contrast, the C1 cluster had significant downregulation of TGFα and Wnt signaling together with processes associated with mesenchymal cell proliferation, proteolysis, cellular transport and RNA metabolism and processing, whereas both the C2 and C1 clusters had decreased representation of processes associated with transcription and splicing. As observed for the upregulated gene processes, the overlap between downregulated gene processes was also low (Additional file 6: Figure S3B).

**Figure 2:**
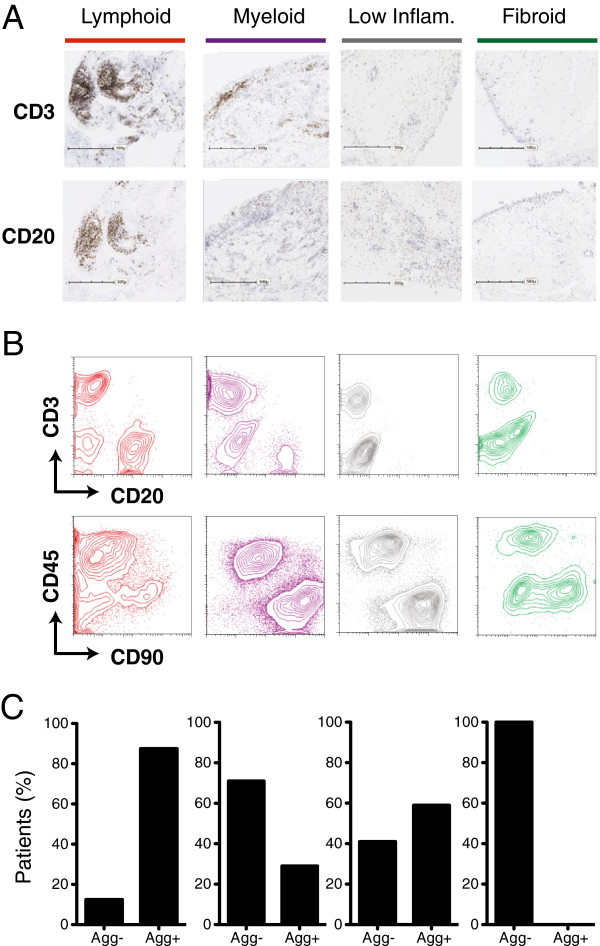
**Rheumatoid arthritis (RA) molecular phenotypes reflect cellular and biological differences. (A)** Immunohistochemical detection of T cells (CD3) and B cells (CD20) in synovial tissue sections. Columns correspond to representative sections for each of the RA molecular phenotypes designated by color-coordinated bars on top. Scales on images refer to a length of 500 microns. **(B)** Fluorescence activated cell-sorting analysis of fresh synovial tissue samples. Cells were stained with CD3- and CD20- gated by forward and side-scatter lymphocyte parameters and fluorescent intensities plotted in a scatter-plot with T cells (CD3) on the y-axis and B cells (CD20) on the x-axis (top panel). Contour-plots from the same patients above showing macrophages (CD45+, lymphocyte-gate exclusion) along the y-axis and fibroblasts (CD90) along the x-axis (bottom panel). Samples are arranged left to right according to their phenotype membership as in panel **A**. **(C)** Bar plots of the percentages of patient synovial tissues that contained non-aggregated (Agg-) or aggregated (Agg+) cellular infiltration as determined by immunohistological assessment of CD3- and CD20-positive cells.

Next, we assessed histological specimens, derived from the tissues used for microarray analysis, for cellular composition and the presence of cellular aggregates reflective of local B and T cell proliferation and lymphoid neogenesis. Representative tissue sections for each cluster were stained with cell-type-specific markers for T cells (CD3) and B cells (CD20) to assess the lymphocyte content of samples (Figure 2A). The results corroborated cellular differences observed in their respective gene-expression profiles. Samples in the lymphoid cluster were enriched for CD20-positive B cells, whereas CD3-positive T cells were present at varying levels in samples from all the major clusters. Using fluorescence-activated cell sorting (FACS) analysis of representative dissociated synoviocyte samples from each cluster (Figure 2B) we found fibroblasts (CD45-/CD90+), macrophages (CD45+/CD90-) and T cells (CD3+) to varying degrees in all clusters, whereas B cells (CD20+) were restricted to lymphoid and myeloid clusters, but were more abundant in lymphoid. Further, histologic cellular aggregates reflecting proliferating B and T cells were abundant in lymphoid samples, present but less abundant in myeloid and low inflammatory samples, and absent in the fibroid samples (Figure 2C).

### Assessment of gene expression and gene sets in RA synovial clusters

**Figure 3:**
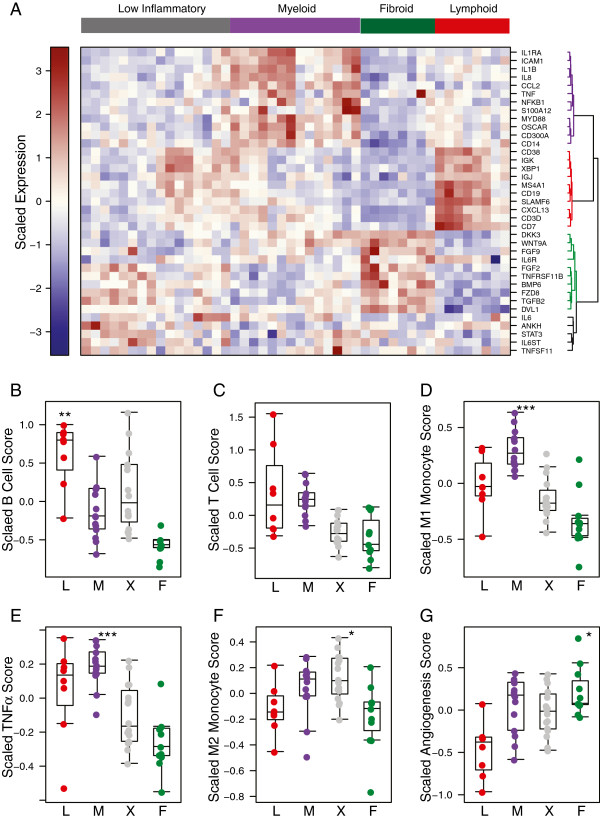
**Distribution of biological process genes and gene sets across the synovial tissue phenotypes. (A)** Heatmap of expression of selected genes in lymphoid (red), myeloid (purple) and fibroid (green) patient subgroups. Patient-sample clusters are supervised by prior phenotype assignment, and genes are distributed by unsupervised clustering. **(B-G)** Distribution of biological processes for each synovial phenotype (L = lymphoid, M = myeloid, X = low inflammatory, F = fibroid) was assessed using predefined gene sets to interrogate the respective microarray datasets. Gene sets reflecting B cells **(B)**, T cells **(C)**, M1 classically activated monocytes **(D)**, genes induced by TNFα **(E)**, M2 alternatively activated monocytes **(F)** and angiogenesis **(G)**. Each subgroup was compared to all other groups using the *f*-test, and significant Benjamini-Hochberg-corrected *P*-values for a group compared with all other groups are indicated (*P ≤0.05, **P ≤0.01, ***P ≤0.001) for subgroups with positive *t*-statistic values.

To further assess the underlying cellular and pathway representation of the identified RA synovial phenotypes, we examined the expression of genes with well-understood biological function that showed differential expression across the RA phenotypes (Figure 3A). The myeloid phenotype had the highest amongst the synovial subgroups of levels of nuclear factor kappa-light-chain-enhancer of activated B cells (NF-κB) pathway genes, including TNFα, IL-1β, IL-1RA, ICAM1, and MyD88, the inflammatory chemokines CCL2 and IL-8, and granulocyte and inflammatory macrophage lineage genes such as S100A12, CD14 and OSCAR. In contrast, the lymphoid phenotype had the highest expression of B cell- and plasmablast-associated genes including CD19, CD20, XBP1, immunoglobulin heavy and light chains, CD38 and CXCL13. The fibroid phenotype had low or absent expression of these genes and instead had elevation of genes associated with fibroblast and osteoclast/osteoblast regulation such as FGF2, FGF9, BMP6, and TNFRSF11b/osteoprotogerin. In addition, this phenotype had higher expression of components of the Wnt and TGFβ pathways. The low inflammatory phenotype showed expression of genes associated with all of the previous phenotypes, indicating this contains representation of all of the prior phenotypes. In addition, expression of IL-6, the IL-6 receptor components IL-6R and IL-6ST/gp130, and associated signaling component STAT3 was broadly observed across all phenotypes, consistent with the multiple roles of the IL-6 pathway in both lymphocyte and fibroblast biology [32].

We further assessed biological processes associated with the synovial phenotypes using experimentally derived gene-set modules representing a spectrum of hematopoietic lineage cells derived from specific expression in purified classically activated M1 monocytes, alternatively activated M2 monocytes, B cells, T cells, TNFα-stimulated synovial fibroblasts and angiogenesis-associated genes (see Methods and Additional file 3: Table S1 for a list of the module genes). The lymphoid phenotype was enriched specifically for B-cell modules (Figure 3B) whereas the myeloid phenotype was enriched for inflammatory M1 monocytes and TNFα-induced modules (Figure 3D, E). In contrast, T-cell genes were expressed similarly in both lymphoid and myeloid phenotypes (Figure 3C). The M2 monocyte module was expressed most highly in the low inflammatory phenotype (Figure 3F) while the angiogenesis module was highest in the fibroid phenotype and lowest in the lymphoid phenotype (Figure 3G). Application of the M1-monocyte and B-cell gene sets to two additional RA synovial datasets showed consistent differential expression patterns to those observed in the initial training dataset, further indicating that these molecular axes define a large proportion of the transcriptional heterogeneity found in the RA synovium (Additional file 7: Figure S4). Further, patients with lower levels of B cell and M1 monocytes had increased levels of fibroid subset genes consistent with the pattern seen in the training data set (Additional file 7: Figure S4B-D). Further, survey of tissue sections characterized by high or low levels of B lymphocytes determined by immunohistochemistry compared with the magnitude of a B-cell gene-set score demonstrated the correlation between histology and gene-set data (Additional file 8: Figure S5). These gene expression data support the notion that there are at least two inflammatory axes of disease in the RA synovium comprising activation of B cells and activation of inflammatory monocytes that are not completely overlapping, whereas other synovial tissues display a low inflammatory pauci-immune phenotype with potential angiogenic, osteoclast/osteoblast dysregulation and fibroblast activation processes in action. Consistent with lack of immune system involvement in the fibroid synovial phenotype, we observed that, for the patients who had available data on serological status, 100% of lymphoid- and myeloid-phenotype patients were RF-positive, 75% of the low inflammatory phenotype patients were RF-positive, and in contrast the fibroid phenotype patients were RF-negative.

### Clinical response to targeted therapies

**Figure 4:**
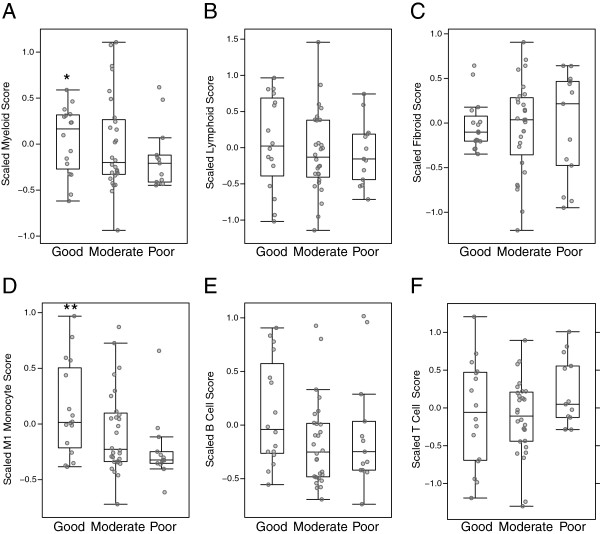
**Pretreatment magnitude of gene sets derived from the synovial myeloid phenotype and classically activated monocytes correlates with clinical response to anti-TNFα (infliximab) therapy.** Analysis of synovial tissue microarray data from 62 rheumatoid arthritis patients in GSE21537 prior to initiation of infliximab (anti-TNFα therapy). Scores for gene sets for phenotypes, defined from the Michigan cohort training data, as well as gene sets derived from purified immune cell lineages (see Methods), were calculated from the GSE21537 data and compared against anti-TNFα clinical outcome at 16 weeks as defined by European League Against Rheumatism (EULAR) response criteria as assigned in GSE21537. Scores versus EULAR response are plotted for the synovial myeloid phenotype **(A)**, lymphoid phenotype **(B)**, fibroid phenotype **(C)**, as well as classically activated M1 monocytes **(D)**, B cells **(E)** and T cells **(F)**. Statistical significance for good compared with poor EULAR response for the level of each gene-set module was calculated based upon the *t*-statistic (* = *P* ≤0.05, **P ≤0.01).

Given the over-representation of myeloid and TNFα-associated gene expression in the myeloid phenotype, we hypothesized that patients who displayed this inflammatory synovial phenotype would have the best clinical response to anti-TNFα treatment as compared with the inflammatory lymphoid phenotype. To test the ability of these predefined synovial phenotypes to identify therapeutic response to TNFα blockade, we interrogated a patient cohort synovial gene-expression dataset (GSE21537 [15], a study that used the anti-TNFα agent infliximab) using pre-specified myeloid and lymphoid gene sets that were derived using an unbiased statistics-based approach from the training cohort data described in Figures 1, 2 and 3 (see Methods). The GSE21537 dataset used a different, non commercial, microarray platform in contrast to the Affymetrix platform utilized for the training set, which required the predefined phenotype gene sets to be mapped onto the GSE21537 microarray expression dataset. Baseline gene-set scores were compared against patient subgroups defined by their EULAR clinical response (good versus poor) to anti-TNFα treatment based upon improvement in the disease activity score from 28 joints (DAS28) at 16 weeks. Strikingly, we observed that baseline expression of the myeloid gene set was significantly higher in patients with good EULAR response compared to non responders (*P* = 0.011, Figure 4A). In contrast, the lymphoid gene set, despite also marking inflammatory synovial processes, did not show association with clinical outcome (*P* = 0.26, Figure 4B) and the fibroid phenotype gene set was also unaltered between good and poor responders (*P* >0.5, Figure 4C).

These results were further confirmed by additional analysis of this dataset using the previously utilized gene sets, which showed that the pretreatment biological process most strongly associated with good versus poor response to anti-TNFα therapy was classically M1 activated M1 monocytes (*P* = 0.006, Figure 4D), whereas in contrast neither the B-cell or T-cell gene sets showed no significant association with response (Figure 4E and F, *P* = 0.18 and *P* = 0.9 respectively). We further observed trends in association of pretreatment levels of M2 alternatively activated monocytes (*P* = 0.054, Additional file 9: Figure S6A) and TNFa-treated synovial fibroblasts (*P* = 0.08, Additional file 9: Figure S6B), whereas angiogenesis processes were significantly associated with good response (*P* = 0.018, Additional file 9: Figure S6C). In addition, we conducted ROC analysis of the gene sets versus EULAR response, and calculation of the AUC revealed that, consistent with the above findings, the myeloid and M1 classically activated monocyte gene sets produced the largest AUCs (0.65, Additional file 10: Figure S7A; and 0.77, Figure S7D respectively). These data indicate that application of predefined molecular synovial phenotypes, namely the myeloid phenotype and associated M1-activated monocytes, has the potential to enrich for responders to anti-TNFα therapy and that pretreatment levels of these biological processes were most strongly associated with anti-TNFα therapeutic outcome.

### Derivation of serum biomarkers from differential synovial gene expression

**Figure 5:**
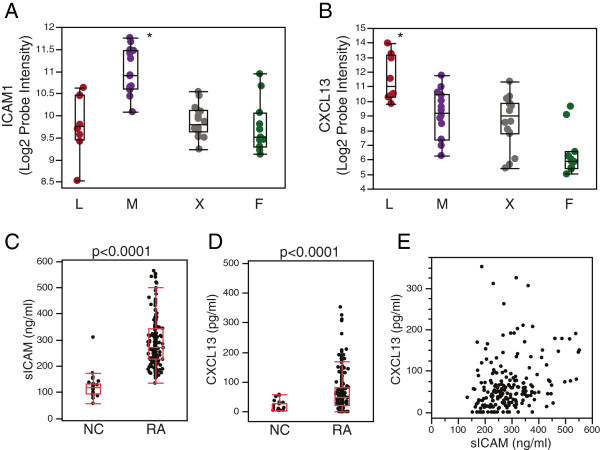
**Assessment of serum biomarkers extrapolated from lymphoid and myeloid synovial phenotype gene expression in the synovial transcriptome training dataset.** Intercellular adhesion molecule 1 (ICAM1) **(A)** and C-X-C motif chemokine 13 (CXCL13) **(B)** genes are expressed at highest levels in the myeloid (M) and lymphoid (L) phenotypes respectively. Array probes for each transcript were compared across all groups using the *f*-test, and in both cases Benjamini-Hochberg-corrected, *P < 0.001. X = low inflammatory phenotype and F = fibroid phenotype. Soluble (s)ICAM1 **(C)** and CXCL13 **(D)** are elevated in serum samples from rheumatoid arthritis (RA) patients (ADACTA trial) as compared with normal control (NC) serum. *P*-values derived from the Wilcoxon test are indicated. **(E)** Serum sICAM1 and CXCL13 levels were only weakly correlated in RA (*ρ* < 0.33, Spearman rank correlation coefficient).

Given the observation that synovial heterogeneity affects treatment outcome to anti-TNFα therapy, we investigated whether we could identify differential gene expression in the inflammatory synovial phenotypes that might be reflected as circulating biomarkers in peripheral blood. Using the *F*-test on the original synovial gene-expression dataset, we identified genes that differed between the synovial phenotypes, and then identified genes that best differentiated one synovial phenotype compared with all others using the pairwise *t*-test between all pairs of groups (*P* <0.001, multiple-hypothesis test correction using the Benjamini-Hochberg method), and further assessed genes encoding potential soluble biomarkers with a positive *t*-statistic value in each phenotype. We focused on two biomarkers: ICAM1, differentially expressed in the myeloid phenotype (Figure 5A), and CXCL13, enriched in the lymphoid phenotype (Figure 5B).

We developed immunoassays to determine levels of circulating soluble ICAM1 (sICAM1) and CXCL13 in serum, and tested pretreatment samples from patients with active RA enrolled in the ADACTA trial (below). We observed that both serum biomarkers were significantly higher in disease compared with samples from non-disease control donors (Figure 5C, D) but importantly were only weakly correlated with each other (Spearman *P* <0.33, Figure 5E) suggesting they are reflective of different inflammatory immune processes.

### sICAM1 and CXCL13 define RA subpopulations with differential clinical outcomes to adalimumab (anti-TNFα compared with tocilizumab (anti-IL-6R) therapy

We finally assessed whether baseline levels of sICAM1 and CXCL13 were differentially associated with subsequent treatment outcome to adalimumab compared with tocilizumab, as we hypothesized based upon the previous results that a population with elevated levels of a myeloid biomarker have elevated clinical response to anti-TNFα therapy but that elevation of a lymphoid marker would not. We utilized pretreatment samples from the ADACTA trial, a randomized, double blind, controlled phase-4 head to head study of tocilizumab (a humanized monoclonal antibody that binds to membrane-bound and soluble forms of the human IL-6 receptor) monotherapy, compared with adalimumab (a fully human monoclonal antibody against TNFα) monotherapy, in methotrexate-intolerant patients with active RA [30]. This trial was notable as it allowed an initial assessment of biomarker-defined populations within the same trial against two different targeted therapies. As this was a post hoc exploratory analysis without pre-specified biomarker thresholds, we first assessed each biomarker individually using the median as a cutoff to define biomarker-low and biomarker-high subpopulations.

**Figure 6:**
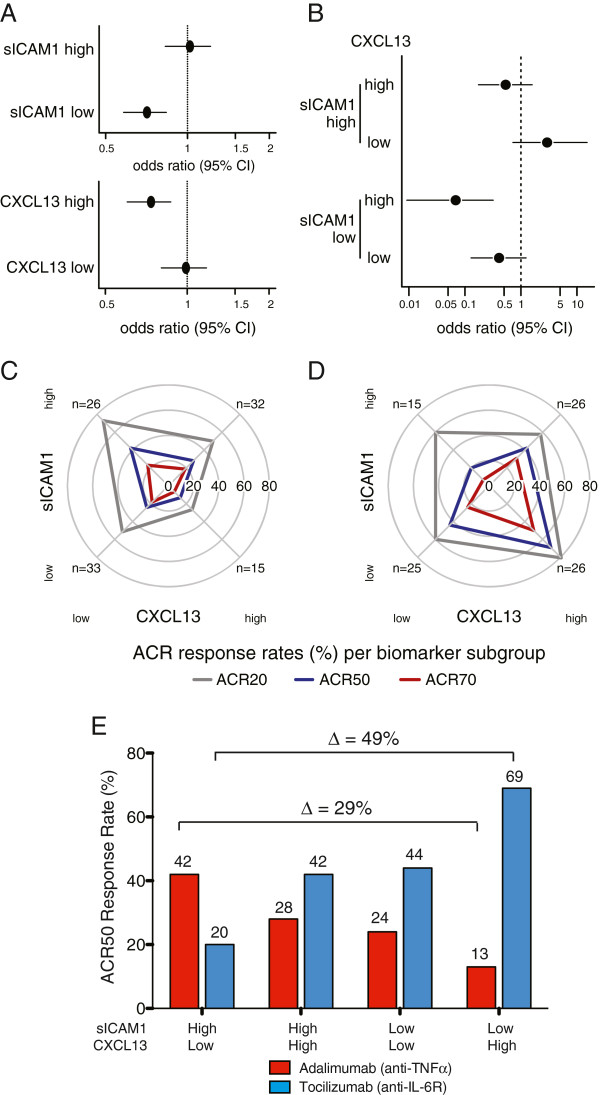
**Lymphoid (C-X-C motif chemokine 13 (CXCL13)) and myeloid (soluble intercellular adhesion molecule 1 (sICAM1)) serum biomarkers define rheumatoid arthritis patient subgroups with differential clinical response to anti-TNFα (adalimumab) compared with anti-IL-6R (tocilizumab) in the ADACTA trial.** Relative treatment effectiveness (week-24 American College of Rheumatology (ACR)50 response) of adalimumab compared with tocilizumab was assessed by logistic regression for **(A)** each individual biomarker and **(B)** biomarker combination-defined subgroups using their respective medians as cutoffs (see Methods). Relative treatment effectiveness for adalimumab versus tocilizumab is represented by odds ratio and 95% CI for ACR50 response. Week-24 ACR20 (gray), ACR50 (green), and ACR70 (purple) response rates (%) per biomarker-defined subgroup are represented by radial plot for adalimumab **(C)** and tocilizumab **(D)** treatment arms. The direction of each radial line corresponds to a biomarker subgroup as follows: sICAM1 low (bottom) and high (top), CXCL13 low (left) and high (right). Low and high designations refer to biomarker values above and below their respective medians. Distance from radial plot center indicates response rate. Summary of week-24 ACR50 response rates for sICAM1-high/CXCL13-low, sICAM1-high/CXCL13-high, sICAM1-low/CXCL13-low and sICAM1-low/CXCL13-high ADACTA RA patients **(E)**. The treatment-effect deltas between sICAM1-high/CXCL13-low and sICAM1-low/CXCL13-high patient groups are indicated for both adalimumab and tocilizumab.

An additional motivation to employ categorical analysis of predictor variables stemmed from the presence of left-censored (below the lower limit of quantification (LLOQ)) observations for baseline levels of CXCL13 where 9.6% (19 of 198 samples) were observed to have values lower than the LLOQ, and categorical analysis was used to accommodate left-censored data, and avoided potential bias that may result from imputation of left-censored data in parametric analyses. We initially observed that there was a differential relationship between clinical outcome to each therapy and baseline biomarker levels: patient populations with lower sICAM1 levels, the myeloid phenotype biomarker, or higher CXCL13 levels, the lymphoid phenotype marker, were associated with lower likelihood, as defined by the odds ratio, of week-24 ACR50 response to adalimumab compared with tocilizumab (Figure 6A). Given these reciprocal associations, we next looked at the two biomarkers in combination, both using the biomarker median values for each as cutoffs as well as continuous biomarker values. These analyses further indicated that heterogeneous treatment effects were present, as the patient population with high sICAM1 but low CXCL13 had higher likelihood of ACR50 response to adalimumab compared with tocilizumab, whereas conversely there was a higher likelihood of ACR50 response to tocilizumab compared with adalimumab in patients with high CXCL13 but low sICAM1 (Figure 6B). Importantly, the differences in relative treatment effectiveness among biomarker-defined subgroups were borne out by contrasting absolute ACR responses among both treatment arms (Figure 6C, D) as opposed to heterogeneous responses observed only in a single treatment arm. Assessing each drug treatment arm separately, using week-24 ACR20, ACR50 and ACR70 response-rates across biomarker median-defined patient subgroups, showed that sICAM1-high/CXCL13-low patients had the highest clinical responses from adalimumab treatment (Figure 6C, E) compared to the other patients in the treatment arm (ACR20 ∆ = 46%, *P* = 0.005; ACR50 ∆ = 29%, *P* = 0.05; and ACR70 ∆ = 16%, *P*-value not significant (Fisher exact test)). Conversely the sICAM1-low/CXCL13-high patients had the highest responses to tocilizumab (Figure 6D, E; ACR20 ∆ = 20%, *P*-value not significant; ACR50 ∆ = 49%, *P* = 0.004; and ACR70 ∆ = 45%, *P* = 0.004 (Fisher exact test)). In addition, the remaining biomarker-defined subgroups (high/high and low/low) exhibited intermediate ACR50 response rates for both therapies (Figure 6E). These differences were also consistent in the trends for change in DAS28-erythrocyte sedimentation rate (ESR) (± standard error) at 24 weeks for adalimumab (-2.3 ± 0.37 for sICAM1-high/CXCL13-low patients compared with -1.1 ± 0.33 for sICAM1-low/CXCL13-high patients) and tocilizumab (-3.6 ± 0.32 for sICAM1-low/CXCL13-high patients compared with -3.2 ± 0.37 for sICAM1-high/CXCL13-low patients). The biomarker-defined subgroup efficacy results for each therapy, including odds ratios for ACR50 response, are summarized in Table 1.

**Table 1:** Summary of baseline biomarker-defined subgroup efficacy at 24 weeks in the ADACTA trial

**Biomarker subset, number**	**ADA ACR20 (%)**	**ADA ACR50 (%)**	**ADA ACR70 (%)**	**ADA ∆DAS28-ESR (±SE)**	**ACR50 odds ratio ADA versus TCZ (95% CI)**
sICAM1^high^/CXCL13^low^ (26)	73	42	23	-2.3 (±0.37)	2.93 (0.7-15.2)
sICAM1^low^/CXCL13^high^ (15)	27	13	7	-1.1 (±0.33)	0.07 (0.009-0.3)
sICAM1^high^/CXCL13^high^ (32)	50	28	19	-2.1 (±0.31)	0.53 (0.17-1.6)
sICAM1^low^/CXCL13^low^ (33)	52	24	18	-2.1 (±0.32)	0.41 (0.13-1.2)
**Biomarker subset, number**	**TCZ ACR20 (%)**	**TCZ ACR50 (%)**	**TCZ ACR70 (%)**	**TCZ ∆DAS28-ESR (±SE)**	**ACR50 odds ratio TCZ vs. ADA (95% CI)**
sICAM1^high^/CXCL13^low^ (15)	60	20	7	-3.2 (±0.37)	0.34 (0.07-1.4)
sICAM1^low^/CXCL13^high^ (26)	81	69	50	-3.6 (±0.32)	14.6 (3.1-108.9)
sICAM1^high^/CXCL13^high^ (26)	58	42	31	-3.2 (±0.37)	1.9 (0.63-5.73)
sICAM1^low^/CXCL13^low^ (25)	60	44	24	-2.9 (±0.36)	2.5 (0.8-7.8)

Data are shown for American College of Rheumatology (ACR) 20, 50 and 70 response rates, change in disease activity score in 28 joints (DAS28)-erythrocyte sedimentation rate (ESR) (± standard error, SE), and odds ratio with 95% CI for ACR50 response. ADA, adalimumab (anti-TNFα); TCZ, tocilizumab (anti-IL-6R).

sICAM1 and CXCL13 biomarker populations were defined by cutoffs determined by the median values. We explored the heterogeneity of the relative treatment effect using alternative biomarker cutoffs using STEPP analysis. Assessment of individual biomarkers showed that increasing levels of sICAM1 were associated with increasing likelihood of ACR50 response to adalimumab versus tocilizumab (Additional file 11: Figure S8A) but increasing levels of CXCL13 were associated with decreasing ACR50 response to adalimumab versus tocilizumab (Additional file 11: Figure S8B). Further, examination of continuous levels of both biomarkers using two-dimensional STEPP analysis also showed the highest likelihood of ACR50 response to adalimumab versus tocilizumab in patients with the highest levels of sICAM1 but the lowest levels of CXCL13 (Additional file 11: Figure S8C), whereas conversely, the lowest likelihood of response to adalimumab versus tocilizumab was observed in the patient population with the lowest sICAM1 and highest CXCL13 levels. These data suggest that further differentiation of relative treatment effect may be observed using optimized cutoffs, as determined in a prospective study.

Finally, ROC analysis was performed to assess the predictive ability for ACR50 response of these two biomarkers on an individual patient basis. sICAM1 and CXCL13 showed only modest predictive ability for adalimumab or tocilizumab on an individual patient basis based upon their respective AUCs (0.57 and 0.6 respectively, Additional file 12: Figure S9A, D), whereas assessment of the two biomarkers in combination showed slight increases in the respective AUCs (Additional file 12: Figure S9C, D, E, F).

In totality, these data illustrate the concept that myeloid and lymphoid phenotype-derived circulating biomarkers can together define RA patient subpopulations that show differential clinical response to therapies directed at different targets, and that myeloid-dominant patient populations with high levels of sICAM1 and low levels of CXCL13 had the most robust response to anti-TNFα therapy.

## Discussion

In this report, we describe the presence of major cellular and molecular heterogeneity in RA synovial tissue characterized by two inflammatory phenotypes dominated by B cells and plasmablasts (lymphoid) and inflammatory macrophages (myeloid) as well as a low inflammatory pauci-immune phenotype, show that elevation of the myeloid, but not lymphoid axis, in synovial tissue is significantly associated with good clinical outcome to anti-TNFα therapy, and finally show that two systemic biomarkers chosen based on their differential tissue expression between the inflammatory phenotypes, CXCL13 for lymphoid and sICAM1 for myeloid, together define RA patient subpopulations with differential clinical response to anti-TNFα compared with anti-IL-6R therapies.

The concept that important heterogeneity exists in RA synovial tissue both at a histological as well as at a molecular level has been previously illustrated by several seminal studies [8,10,33], which showed differential presence of histological synovial aggregates and diffuse synovial inflammation, as well as differential gene expression, across RA synovial samples. The objective of the current study was to test the idea that heterogeneous RA synovial tissues can be assigned to subgroups that share common patterns of gene expression, have different associated systemic biomarkers, and that might respond differentially to therapy. Thus, we employed an analysis strategy that queried independently the questions of molecular heterogeneity and response heterogeneity. First, we assessed molecular heterogeneity of RA synovium independent of treatment response and validated proposed phenotypes using various molecular techniques and external patient cohorts. We next observed that core biological modules, as defined using pathway analysis, designated lymphoid (B cell- and plasmablast-dominated), myeloid (macrophage and NF-κB process dominated) and fibroid (comprising hyperplastic but pauci-immune tissues) could be surveyed across multiple RA patient synovial tissue cohorts to identify reproducible RA phenotypes. Importantly, the dominant biology associated with each gene expression-defined subset was consistent with histological and flow cytometry assessment of synovial tissue where the lymphoid subset was associated with presence of histological aggregates and, the myeloid subset with more diffuse immune infiltration while the fibroid subset had little immune infiltration and complete absence of aggregates. Further, survey of tissue sections characterized by high or low levels of B lymphocytes determined by immunohistochemistry correlated with the magnitude of a B cell gene-set score. We also observed the presence of a low inflammatory phenotype, indicating that synovial heterogeneity exists as a continuum of dysregulated biological processes rather than absolutely discrete subsets of disease. We did not observe differences in therapeutic usage (methotrexate, anti-TNFα agents, steroids) between patients with different synovial phenotypes where these data were available (data not shown). However, we did note that for the patients with data available, RF serological positivity was restricted to the lymphoid, myeloid and a majority of the low inflammatory phenotype patients. These data are consistent with previously observed gene expression heterogeneity in RA synovial tissue suggesting there are both inflammatory and non inflammatory synovial subgroups in RA. We further observed presence of patients with low or high inflammatory phenotypes based upon M1-activated monocytes, B cell and fibroid gene sets in two additional datasets, although the M1 and B cell gene sets were not as divergent as observed in the original training set. Reasons for this could include introduction of additional noise and loss of sensitivity due to the different platform used in the GSE21537 dataset resulting in loss of data due to missing or non-mapping probes as compared with the Affymetrix platform as well as differences in the patient populations, as there were higher levels of fibroid gene-set scores in both patient cohorts compared with the training dataset, meaning decreased representation of patients in the highly inflammatory subgroups.

Indeed, it has been clearly shown that patients with high levels of expression of inflammatory genes in the synovium have higher levels of systemic inflammation including C-reactive protein levels, ESRs and platelet counts as well as a shorter duration of disease as compared to patients with low synovial inflammation [34]. Further, absence of significant synovial inflammation has been linked to decreased presence of anti-citrullinated protein antibodies [35]. Consistent with this finding of a pauci-immune phenotype of RA, patients with lower levels of both synovial and systemic inflammation have been shown to have lower drug-response rates to both B-cell depletion therapy and anti-TNFα [36-38].

We then assessed whether the inflammatory biological modules would be differentially informative for predicting the outcome of response to anti-TNFα therapy through analysis of a large and well-defined external dataset. Strikingly, patients with high pretreatment expression of genes defined in the myeloid phenotype and M1 classically activated monocytes, but not high levels of lymphoid subset or B-cell genes, showed a greater 16-week good EULAR response to infliximab treatment. This is consistent with the observation that inflammatory M1 macrophages, a key lineage involved in production of TNFα, as well as expression of TNFα itself along with IL-1β and NF-κB-associated processes, are preferentially increased in the myeloid phenotype compared with all of the others. Further, other studies have consistently concluded that baseline levels of synovial macrophages and TNFα gene expression are correlated with response [13,39], suggesting the presence of TNFα-secreting classically activated monocytes and macrophages are important for clinical outcome. However, the EULAR moderate responders had a wide range of values for both the myeloid and M1 genes, which suggest that other factors will contribute to determining treatment outcome with anti-TNFα agents. In contrast, a large histological study demonstrated that RA patients with high levels of synovial lymphoid neogenesis (LN), comprising highly organized B/T cell aggregates, demonstrated resistance to anti-TNFα therapy and good clinical outcome in these patients was accompanied with reversal of LN [40]. Consistent with this, we observed that the presence of the lymphoid phenotype was not a predictor of response to anti-TNFα despite being associated with the presence of synovial inflammation and histological aggregates. In sum, these data suggest that simply the presence of inflammation alone is insufficient to predict clinical outcome to anti-TNFα treatment, and rather that sub-phenotypes of synovitis show differential clinical benefit with the lymphoid phenotype showing greater resistance to anti-TNFα as compared with the myeloid phenotype, perhaps due in part to the presence of other major processes driving synovitis including production of other inflammatory mediators, LN, and robust antigen presentation by autoreactive B cells. It is also noteworthy that we observed an association between pretreatment expression of genes associated with angiogenesis and clinical response to anti-TNFα, suggesting that the presence of synovial neoangiogenesis may also contribute to favorable outcome to blockade of TNFα.

Next, we hypothesized that the biological processes underlying the RA phenotypes might allow for rational serum protein biomarker selection to prospectively identify patient populations prior to starting a targeted therapy. As synovial tissue is not readily available for prospective assessment prior to initiation of therapy, systemic circulating biomarkers have greater potential utility although they will likely integrate the activity of specific biological pathways in multiple tissues, including the secondary lymphoid system, in addition to synovial tissue. We assessed candidates that were differentially expressed in the inflammatory lymphoid and myeloid subsets using a statistical ranking, and looked for markers that were strongly elevated in RA serum as compared with serum from non disease control donors. Two markers that fulfilled these criteria were soluble ICAM1 (myeloid) and CXCL13 (lymphoid). ICAM1, an adhesion molecule that binds to LFA-1, is a gene that is strongly regulated by NF-κB signaling and is upregulated on a variety of cell types in response to TNFα signaling including synovial fibroblasts and especially vascular endothelial cells, both of which are highly represented in the inflammatory rheumatoid synovium [41,42]. sICAM1 is shed from the cell membrane by proteolytic cleavage. CXCL13 is a B cell chemoattractant that is highly expressed by follicular dendritic cells in secondary lymphoid tissue and ectopic germinal centers and is induced by LTα/LTβR signaling [43]. Further, a recent report of a small synovial biopsy study of RA patients undergoing rituximab therapy showed a correlation between synovial tissue expression of CXCL13 and levels of CXCL13 protein in the serum (*r* = 0.6) [44] that suggests CXCL13 expression in the rheumatoid synovium is a major source of serum CXCL13. Synovial and serum levels of CXCL13 have also recently been linked with radiological joint destruction in RA patients [45], which argues that this gene, and by association the lymphoid synovial phenotype, is linked with progressive and destructive RA pathogenesis. In contrast, to our knowledge no reports have been made to date that have directly compared sICAM1 levels in serum with ICAM1 gene expression in synovial tissue, and we have not been able to conduct such an analysis in this study due to incomplete matching serum samples. Analysis of serum samples from the ADACTA adalimumab (anti-TNFα) compared with tocilizumab (anti-IL-6R) trial facilitated an assessment of these biomarkers in an inflammatory RA population that not only allowed a direct comparison of clinical response to different targeted therapies within one clinical study, but also avoided confounding effects of concomitant immunosuppression from background methotrexate as this study was conducted using both therapeutic agents as monotherapy [30]. Consistent with our model of different inflammatory axes being present in RA, we noted that although both sICAM1 (myeloid) and CXCL13 (lymphoid) were significantly elevated in disease compared with control samples, they were only weakly correlated to each other. Further, we noted that patients with high pretreatment serum sICAM1 levels and decreased CXCL13 levels (high myeloid and low lymphoid activity) had increased ACR50 and ACR70 response rates and decreased DAS28-ESR scores to anti-TNFα therapy compared with anti-IL-6R therapy, whereas conversely, patients with high CXCL13 and decreased sICAM1 levels had preferential response to anti-IL-6R compared with anti-TNFα therapy. We did note differences in the magnitude of the differences between ACR50 response rates and changes in DAS28-ESR between the biomarker-defined populations in the tocilizumab arm where the changes in DAS28 were consistent but smaller than those observed for ACR50. These differences could not be accounted for by one component of the response instrument, for example, ESR or swollen-joint count, and are likely due more to differences in precision between the two instruments. These results are consistent with the previous data showing that patients with elevation of the myeloid inflammatory axis had robust responses to anti-TNFα drugs, and further emphasize that within an inflammatory RA population, there are patient subsets that subsequently have differential clinical outcomes to different targeted therapies.

What underlying biological basis could explain why blockade of the IL-6 pathway causes robust clinical responses in a different patient population to that responding to anti-TNFα blockade? Although IL-6 has long been appreciated as a key inflammatory cytokine important in the pathogenesis of RA as well as other inflammatory diseases [32], its biology and expression are not completely overlapping with that of TNFα. Our synovial tissue gene-expression data have shown that although TNFα is strongly associated with the myeloid phenotype and activity of classically activated myeloid cells and NF-κB pathway activity, IL-6, its receptors IL-6R and IL-6ST/gp130, and the key IL-6-associated TF, STAT3, are more broadly expressed across the lymphoid and low inflammatory synovial subsets (Figure 3A) and are not highly correlated with TNFα expression or restricted to the myeloid phenotype. Indeed, IL-6 can be induced in a variety of cell lineages exposed to multiple inflammatory stimuli in the joint including synovial fibroblasts themselves [32,46]. Further, the IL-6/IL-6R pathway signals using the JAK/STAT pathway in contrast to the canonical NF-κB signaling predominantly utilized by TNFα [47] and plays a key role in inducing B cells to differentiate to antibody-secreting cells. Importantly, anti-IL-6R therapy has been shown to be effective in patients who are refractory to anti-TNFα therapies [48]. Thus, it is conceivable that the IL-6/IL-6R pathway is highly involved with the driving synovitis in the B-cell-dominant lymphoid axis as well as potentially similarly important in driving synovitis in the low inflammatory subset, whereas in contrast, within the activated monocyte-dominated myeloid axis the TNFα pathway is dominant in driving synovitis such that blockade of IL-6 signaling is less effective. Whilst intriguing, and consistent with the biological hypotheses developed based upon our synovial tissue analyses, the findings described here represent only an initial testing of the sICAM1/CXCL13 biomarker hypothesis without a predefined cutoff for the analysis, hence our utilization of the median as the cutoff for this analysis, and the statistical power was limited by available patient numbers and multiple testing issues. Furthermore, analysis of these biomarkers on an individual patient basis using ROC analysis showed that they have only modest predictive ability for ACR50 outcome to adalimumab or tocilizumab at 24 weeks. Therefore, although the biomarkers described here demonstrate the presence of populations of RA patients with differential clinical response to targeted therapies, they do not presently have strong clinical utility for decision-making for individual patients. Improvement of individual patient predictive-ability might be achieved by incorporation of additional biomarkers into a predictive model that could be subjected to rigorous confirmatory studies in larger patient cohorts treated with anti-TNFα and anti-IL-6/IL-6R blocking agents including combination treatment with methotrexate, with incorporation of prespecified cutoff values in the analysis plan. Indeed, the two-dimensional STEPP analysis performed in this study suggested that altering the biomarker threshold cutoffs for both sICAM1 and CXCL13 could yield greater efficacy differentials for ACR50 response rates between adalimumab and tocilizumab than those achieved by using their respective medians.

Additional limitations of this study include limited availability of clinical data in the RA cohort used for the initial gene-signature discovery owing to the retrospective nature of interrogation of clinical chart data after sample collection from joint surgery, and a lack of consent for chart review in some cases. In particular, there were incomplete or missing data for serological autoantibody status for RF or anti-citrullinated protein antibodies. Also, the RA patient population studied for synovial gene expression represents late-stage disease where patients received joint surgery to correct deformity, replace joints, or manage pain. This study also does not address the presence and stability of synovial phenotypes longitudinally from early to late-stage disease and with respect to development of bone erosion. Finally, in the current study we have not applied an exhaustive investigation of all the potential serum biomarkers that may correlate with synovial subtypes, in part due to the desire to minimize multiple testing issues due to the limited number of anti-TNFα-treated patient samples available for biomarker analysis. These important questions are being addressed in a series of follow-up prospective studies.

## Conclusions

Utilizing genome-wide expression analysis of synovial tissues from a large RA cohort, we have defined distinct molecular and cellular phenotypes that reflect the considerable heterogeneity present in the RA synovium. In particular, two distinct inflammatory axes emerge from this analysis: one dominated by B cells and the other dominated by inflammatory macrophages and NF-κB-activating cytokines, such as TNFα. It is important to point out that these cellular and molecular signatures, as well as the RA patients, represent a continuous rather than a discrete distribution, as is evident from the presence of lower inflammatory patients with intermediate molecular characteristics between these polar phenotypes. Analysis of respective gene-set modules and serum biomarkers suggest differential clinical response to anti-TNFα and anti-IL6R therapy is dependent in part on the presence of these inflammatory axes. A further subgroup of patients presented with a pauci-immune phenotype lacking major B cell or macrophage infiltration and may reflect a distinct subgroup of patients. These synovial phenotypes explain some of the underlying clinical and drug response heterogeneity in RA, and identifying and stratifying patients prospectively with respect to their synovial phenotype, for example by using blood biomarkers, may be important in making therapeutic decisions for targeting therapies. Such considerations are also likely to be very important for clinical trial design for new therapies to select patients prospectively for increased clinical response rates, and for the design of clinical studies to differentiate targeted therapies with different mechanisms of action.

## Abbreviations

ACR: American College of Rheumatology; ADACTA: ADalimumab ACTemrA; Agg: aggregated; AUC: area under the receiver-operating characteristic curve; BMP: bone morphogenetic protein; CXCL13: C-X-C motif chemokine 13; DAB: 3,3′-diaminobenzidine; DAS28: disease activity score (from 28 joints); DAVID: Database for Annotation Visualization and Integrated Discovery; DMARD: disease-modifying anti-rheumatic drug; ESR: erythrocyte sedimentation rate; EULAR: European League Against Rheumatism; FACS: fluorescence-activated cell sorting; FDR: false discovery rate; HCL: hierarchical clustering; IFN: interferon; IL: interleukin; JAK: Janus kinase; LLOQ: lower limit of quantification; LN: lymphoid neogenesis; LPS: lipopolysaccharide; MSigDB: Molecular Signatures DataBase; NF-κB: nuclear factor kappa-light-chain-enhancer of activated B cells; NS: not significant; NSAID: non-steroidal anti-inflammatory drug; PAM: partitioning around medoids; RA: rheumatoid arthritis; RF: rheumatoid factor; RMA: robust multichip average; ROC: receiver-operating characteristic; sICAM1: soluble intercellular adhesion molecule 1; STAT: signal transducer and activator of transcription; STEPP: subpopulation treatment effect pattern plot; TNF: tumor necrosis factor.

## Competing interests

The studies described here were funded by Genentech/F. Hoffmann-La Roche, the current or former employer of GD, CH, SK, DC, AFS, JH, PH, HG, WYL, LD, SF, AS, DM, MK, FM and MT who participated in study design, data collection, analysis and preparation of the manuscript.

## Authors' contributions

GD: data collection and analysis, manuscript writing, critical revision, final approval of manuscript. CH: data collection and analysis, manuscript writing, critical revision, final approval of manuscript. SK: data analysis, manuscript writing, critical revision, final approval of manuscript. DC: data analysis, critical revision, final approval of manuscript. AFS: data collection and analysis, critical revision, final approval of manuscript. WYL: data collection and analysis, critical revision, final approval of manuscript. LD: data analysis, critical revision, final approval of manuscript. SF: data collection and analysis, critical revision, final approval of manuscript. AS: data collection and analysis, critical revision, final approval of manuscript. JH: data analysis, critical revision, and final approval of manuscript. PH: data analysis, critical revision, and final approval of manuscript. HG: data analysis, critical revision, and final approval of manuscript. DM: data collection, critical revision, and final approval of manuscript. MK: data collection, critical revision, and final approval of manuscript. CG: data collection, critical revision, and final approval of manuscript. AK: data collection, critical revision, and final approval of manuscript. JE: acquisition of samples, data collection and final approval of manuscript. DF: acquisition of samples, data collection and analysis, critical revision, final approval of manuscript. FM: study conception and design, data collection and analysis, manuscript writing, final approval of the manuscript. MT: study conception and design, data collection and analysis, manuscript writing, critical revision, final approval of the manuscript. All authors read and approved the final manuscript.

## Authors’ information

Flavius Martin and Michael J Townsend co-directed the project.

## Supplementary Material

Additional file 1 **Lists of the Database for Annotation, Visualization and Integrated Discovery (DAVID) biological processes genes represented within the upregulated genes in the synovial subgroups.** For each gene, we report differential gene expression between each group and all other samples. We provide the *t*-statistic values (positive or negative) with associated *P*-values for each group. L = lymphoid, M = myeloid, X = low inflammatory, F = fibroid. Click here for file

Additional file 2 **Lists of the Database for Annotation, Visualization and Integrated Discovery (DAVID) biological process genes represented within the downregulated genes in the synovial subgroups.** For each gene, we report differential gene expression between each group and all other samples. We provide the *t*-statistic values (positive or negative) with associated *P*-values for each group. L = lymphoid, M = myeloid, X = low inflammatory, F = fibroid. Click here for file

Additional file 3: Table S1 List of genes utilized in gene set enrichment analyses. Click here for file

Additional file 4: Figure S1 Assessment of robustness of synovial gene expression heterogeneity. **(A)** Principal component analysis showing the first (x-axis) and second (y-axis) components of variation over approximately 7,000 probes and 49 patients using the prcomp R-function on quantile-normalized expression data. Each patient tissue is color-coded according to the groupings in Figure 1A, and grouping circles have been added for visual clarity. **(B)** Re-sampling analysis using partitioning around medoids (PAM) analysis of approximately 7,000 probes, 49 patients and 5 predefined clusters of tissue samples (*k* = 5). Heatmap colors represent the frequency with which a pair of samples are found in the same cluster, and are represented as a percentage of the total number of samplings in which the pair was observed. **(C)** Assessment of cluster robustness via determination of silhouette width of approximately 7,000 clustered probes from the 49 patients. Average silhouette widths for each of the five clusters are indicated. Click here for file

Additional file 5: Figure S2 Assessment of overlap between biological process gene-sets utilized by the Database for Annotation, Visualization and Integrated Discovery (DAVID) pathway analysis tool for unregulated genes in each of the four synovial clusters defined in Figure 1A. The overlap of genes shared by gene sets are illustrated using a heatmap where each value represents the proportion of genes from the category on the y-axis that are in common with the corresponding gene set on the x axis (indicated by the color bar; 0 = 0%, 1 = 100%). The matrix is not symmetrical because the size of the gene sets is not constant. Click here for file

Additional file 6: Figure S3 **(A)** Heatmap visualization of processes enriched in downregulated genes in each of the four synovial clusters defined in Figure 1A using the Database for Annotation, Visualization and Integrated Discovery (DAVID) pathway analysis tool. Colors refer to statistical significance of processes to each cluster. **(B)** Assessment of overlap between biological process gene sets utilized by the DAVID pathway analysis tool for downregulated genes in each of the four synovial clusters defined in Figure 1A. The overlap of genes shared by gene sets are illustrated using a heatmap where each value represents the proportion of genes from the category on the y-axis that are in common with the corresponding gene set on the x-axis (indicated by the color bar; 0 = 0%, 1 = 100%). The matrix is not symmetrical because the size of the gene sets is not constant. Click here for file

Additional file 7: Figure S4 B cell, M1 classically activated monocyte, and fibroid gene modules capture synovial tissue transcriptional heterogeneity in additional rheumatoid arthritis (RA) patient cohorts. **(A)** Scatter plot of the training cohort of 49 patient synovial samples projected in gene set space of the B cell (x-axis) and M1 monocyte (y-axis) biological modules. Samples are colored according to their cluster assignments in Figure 1 (red = lymphoid, purple = myeloid, green = fibroid, grey = low inflammatory). Filled circles indicate samples with histologic aggregates and empty circles indicate samples lacking aggregates. Scatter plot of the same 49 RA patients projected in gene set space of the B cell (x-axis) and M1 monocyte (y-axis) biological modules, and samples are also colored according to their respective fibroid gene set scores as indicated by the color bar. **(C)** Scatter plot of 33 previously unanalyzed patient samples from a parallel Michigan RA cohort projected in gene-set space of the B cell (x-axis) and M1 monocyte (y-axis) biological modules. Samples are colored according to their respective fibroid gene-set scores as indicated by the color bar. **(D)** Scatter plot of a publicly available cohort of 62 RA histologically characterized patients (GSE21537) projected in gene-set space of the B cell (x-axis) and M1 monocyte (y-axis) biological modules. Samples are colored according to their respective fibroid gene-set scores as indicated by the color bar. Click here for file

Additional file 8: Figure S5 CD20 Immunohistochemistry (IHC) correlates with B cell gene-set score in a replication rheumatoid arthritis (RA) patient cohort. Representative CD20 IHC (brown staining) is shown for synovial samples with a high or low B cell gene-set score with low (**A**, **B** respectively) and high (**C**, **D** respectively) magnification. B cell gene-set scores were also plotted against CD20 IHC scores and the *P*-value for Spearman rank correlation coefficient is indicated **(E)**. Click here for file

Additional file 9: Figure S6 Association of pretreatment synovial gene-set scores with good versus poor European League Against Rheumatism (EULAR) response to anti-TNFα (infliximab) therapy at 16 weeks in the GSE21537 synovial expression dataset. Statistical significance for good compared with poor response for the level of each gene-set module was calculated based upon the *t*-statistic. Scaled gene-set scores for M2 alternatively activated monocytes **(A)** (*P* = 0.054), TNFα-stimulated fibroblast-like synoviocytes **(B)** (*P* = 0.08), and angiogenesis **(C)** (*P* = 0.02) marked with asterisk) are plotted against 16-week EULAR response. Click here for file

Additional file 10: Figure S7 Receiver-operating-characteristic (ROC) curves to assess the ability of pretreatment synovial phenotypes, defined by scaled gene-set scores, to differentiate between good versus poor European League Against Rheumatism (EULAR) response to anti-TNFα (infliximab) therapy at 16 weeks in the GSE21537 synovial expression dataset. ROC curves were generated for the myeloid **(A)**, lymphoid **(B)** and fibroid **(C)** phenotypes, and also for gene sets reflective of M1 classically-activated monocytes **(D)**, B cells **(E)** and T cells **(F)**. Area under the ROC curve (AUC) is indicated for each plot. Click here for file

Additional file 11: Figure S8 Biomarker subpopulation treatment effect pattern plot (STEPP) analysis of the ADalimumab ACTemrA (ADACTA) trial. Assessment of individual biomarkers compared with treatment effect. One-dimensional STEPP analysis of week-24 American College of Rheumatology (ACR) 50 relative treatment effectiveness of adalimumab compared with tocilizumab for the serum markers soluble intercellular adhesion molecule 1 (sICAM1) **(A)** and C-X-C motif chemokine 13 (CXCL13) **(B)** respectively in the ADACTA trial. Week-24 ACR50 odds ratios are shown in solid blue and 95% CIs as accompanying dashed lines. The x-axes correspond to the subgroup of subjects whose baseline biomarker levels were within 20 percentiles below and above the indicated subpopulation median with actual values (pg/ml) in parentheses. The dotted horizontal line indicates equivalent relative treatment effect. **(C)** Two-dimensional STEPP analysis for sICAM1 and CXCL13. Each cell of the heatmap corresponds to a subgroup of subjects whose baseline biomarker levels were within 25 percentiles below and above the indicated subpopulation median as defined by each biomarker. Concentrations of each biomarker at the indicated percentage are in parentheses in plot margins. Heatmap colors indicate odds ratio (95% CI in brackets) from logistic regression corresponding to outcomes for adalimumab versus tocilizumab. Counts of subjects in each treatment arm for each subgroup are indicated as n = (tocilizumab)/(adalimumab). Click here for file

Additional file 12: Figure S9 Receiver-operating-characteristic (ROC) curves to assess the ability of pretreatment C-X-C motif chemokine 13 (CXCL13) and soluble intercellular adhesion molecule 1 (sICAM1) to differentiate for clinical response in the ADalimumab ACTemrA (ADACTA) trial biomarker population. ROC curves were generated for sICAM1 versus achievement of an American College of Rheumatology (ACR)50 response at week 24 for adalimumab in all-comers **(A)**, CXCL13-high **(B)**, and CXCL13-low patient subsets **(C)**, and for CXCL13 versus achievement of an ACR50 response at week 24 for tocilizumab in all-comers **(D)**, sICAM1-high **(E)** and sICAM1-low patient subsets **(F)**. Biomarker high and low designations were made using their respective medians as the cutoff. Area under the ROC curve (AUC) is indicated for each plot. Click here for file
